# Increased Crown-To-Implant Ratio May Not Be a Risk Factor for Dental Implant Failure under Appropriate Plaque Control

**DOI:** 10.1371/journal.pone.0063992

**Published:** 2013-05-30

**Authors:** Shinsuke Okada, Katsunori Koretake, Yasunari Miyamoto, Hiroshi Oue, Yasumasa Akagawa

**Affiliations:** 1 Department of Advanced Prosthodontics, Graduate School of Biomedical and Health Sciences, Hiroshima University, Hiroshima, Japan; 2 Department of Advanced Prosthodontics, Institute of Biomedical and Health Sciences, Hiroshima University, Hiroshima, Japan; 3 Department of Advanced Prosthodontics, Institute of Biomedical and Health Sciences, Hiroshima University, Hiroshima, Japan; University of Toronto, Canada

## Abstract

**Objective:**

The aim of this study was to evaluate whether increased crown-to-implant (C/I) ratio influences implant stability or not under proper healthy control of peri-implant mucosa. The hypothesis of this study is that implant stability can be maintained despite High C/I, under appropriate plaque control.

**Materials and Methods:**

Five male Beagle-Labrador hybrid dogs (2 years old) were used. Their bilateral mandibular premolar extraction was performed. After allowing 12 weeks for bone healing, 3 types of vertical marginal bone loss were simultaneously prepared randomly. Then, 30 titanium implants were placed in the edentulous areas and defined as High C/I, Mid C/I and Low C/I groups. This time point was designated as the baseline (0 Week). Twelve weeks after implant placement, metal superstructures were cemented to the implants and an occlusal plate was set at the opposite side. At the same time, Calcein green was injected for remodeling evaluation. Implants were loaded by feeding the dogs a hard pellet diet. Tooth brushing was performed 5 days per week during the study to maintain healthy peri-implant mucosa. Twenty-four weeks following implant placement, the interface structure was evaluated clinically, radiologically, and histologically.

**Result:**

Implant stability quotient (ISQ) increased with time in all 3 groups, without any significant correlation with the C/I value (*p* **>**0.05). Moreover, mean marginal bone loss adjacent around implants in all 3 groups ranged between 0.11 and 0.19 mm, with no significant difference (*p* **>**0.05). Many fluorescence-labeled bones are shown in the High C/I group. It is considered that high remodeling activity prevent marginal bone loss in the High C/I group and this may provide favorable implant stability under proper plaque control.

**Conclusion:**

These findings suggest that increased C/I may not be a risk factor for implant failure if the peri-implant mucosa is kept healthy, as was the case in this animal model.

## Introduction

Osseointegration [1] is defined as direct contact between bone and implant with no soft tissue intervention, and is regarded as a prerequisite for long-term implant success [2], [3]. The criteria for implant success were defined at a conference in Toronto in 1998 [4] : individually unattached implants should be immobile when tested clinically and mean vertical bone loss should be <0.2 mm 12 months following implantation. However, in recent years, peri-implantitis has come to be regarded as a major factor for loss of osseointegration, resulting in implant failure. Excessive peri-implant bone loss leads to a higher crown-to-implant (C/I) ratio, thus altering the biomechanics of the implant [5]. C/I ratio is defined as the physical relationship between individual restorative items located both within and outside the bone [6]. Misch [7] reported that a C/I ratio of 0.5–1.0 reduces stress on the peri-implant bone, thereby preventing bone loss and implant failure. On the other hand, Tawil *et al*. [8] reported no correlation between C/I ratio and marginal bone loss. However, the above results regarding C/I ratio were obtained via nonstandardized clinical studies, and no biological reactions were observed *in vivo* study. We are awaiting clinical criteria evaluating the biomechanics of implantation with increased C/I ratio. Of late, it has become common to clinically evaluate implant stability by measuring the implant stability quotient (ISQ) value [9]. Becker *et al*. [10] reported that a high initial ISQ following implantation often dropped slightly over time, while the frequency of ISQ levels <60 increased between implant insertion and abutment connection. Scarano *et al*. [11] reported statistically significant correlation between ISQ and the implant–bone interface in humans. Nedir *et al*. [12] reported that implant stability could be reliably determined for implants displaying an ISQ >47. The hypothesis of this study is that implant stability can be maintained despite High C/I, under appropriate plaque control. If this hypothesis can be proved, a clinical index based on C/I ratio related to implant biomechanics can be established. The aim of this study was to evaluate whether higher C/I values influence implant stability while maintaining an appropriately healthy status of the peri-implant mucosa.

## Materials and Methods

### Ethics Statement

The animal research protocol was in accordance with the current version of the Japan Law on the Protection of Animals. This study was approved by the Research Facilities Committee for Laboratory Animal Science at the Hiroshima University School of Medicine, Hiroshima, Japan. All surgery was performed under general anesthesia, and all efforts were made to minimize suffering during experimental period. We purchased the dogs from Hiroshima Laboratory of Experimental Animals. All dogs in our study were Beagle Labrador hybrid dogs and they were domestic. The owners of the dogs gave permission for their animals to be used in our study.

**Figure 1:**
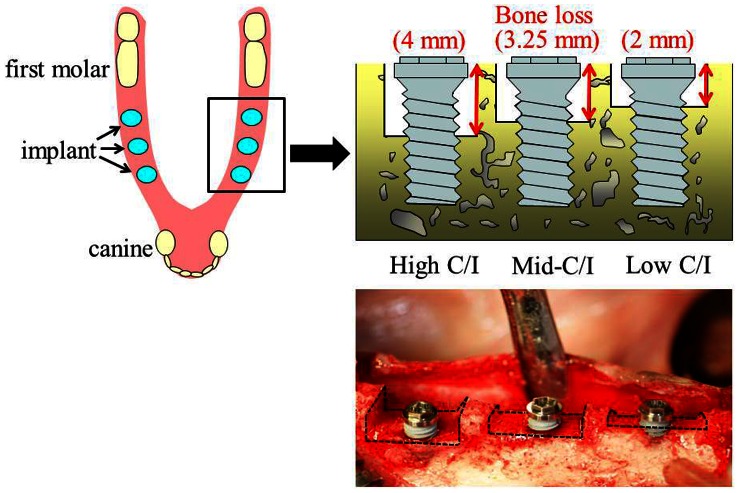
Three types of C/I with prepared different bone loss.

**Figure 2:**
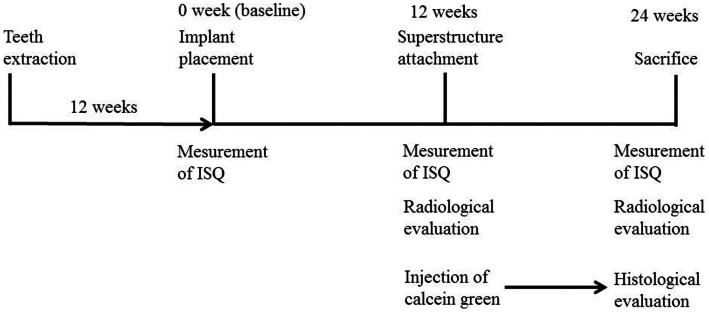
Design of the animal experiment.

Five male Beagle-Labrador hybrid dogs (2 years old) were used. Bilateral extraction of the animals’ mandibular premolars (P1-P4) was performed, and the area was allowed to heal for 12 weeks to prepare the edentulous area. Then, the residual ridge was flattened (mx-grafter®, Maxilon Laboratories, Inc., Hollis, NH, USA) by cutting bone so that the marginal bone level coincided bucco-lingually. After allowing 12 weeks for bone healing, vertical bone loss was simultaneously prepared randomly in 3 simulated C/I situations (High, 4 mm; Mid, 3.25 mm; Low, 2 mm) using a special electric motor (Nobel Biocare Japan Inc., Tokyo, Japan). With the use of a series of drills and screw taps (Nobel Biocare Japan Inc., Tokyo, Japan), 30 titanium implants (Brånemark® Ti Unite Mark III, diameter 3.75 mm, length 7.00 mm; Nobel Biocare, Sweden) were placed in these edentulous bone loss areas and defined as High C/I, Mid C/I and Low C/I (Fig. 1). This time point was designated as the baseline (At 0 Week). ISQ values were measured by a wireless Osstell device (Osstell® Mentors; Integration diagnostics AB, Göteborg, Sweden) to evaluate implant stability at 0, 12, and 24 weeks after implant placement. These measurements were carried out twice each in 2 perpendicular directions (mesio-distal and bucco-lingual), and the mean values were calculated (Fig. 2). An imaging jig and a dental imaging indicator (Imaging Indicator II^®^; Hanshin Technology Laboratory) were fixed bilaterally to the canines, and standardized radiographs were taken to evaluate the bone interface at 0, 12, and 24 weeks after implant placement.

**Figure 3:**
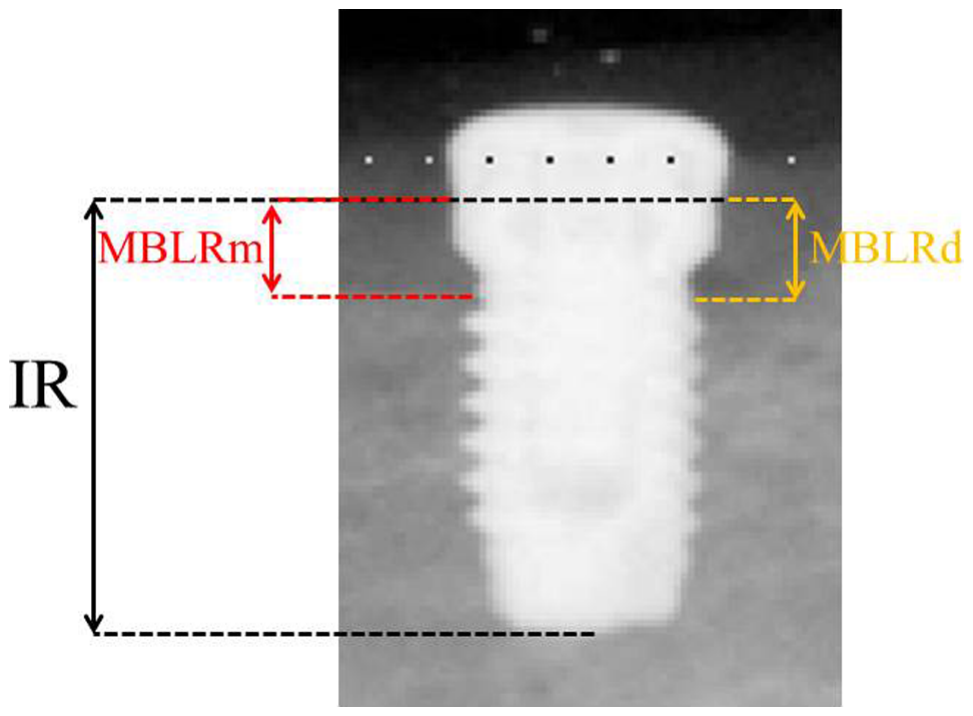
Superstructure constructed from a gold–silver–palladium alloy using an articulator.

**Figure 4:**
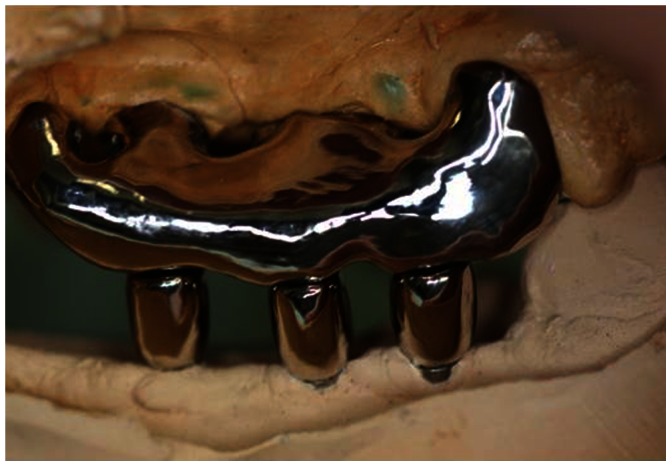
Superstructures are attached to the lower implants at a height of 8 mm. The occlusal plate is attached to the maxillary molars to maintain occlusal contact without any lateral loadings by the superstructures.

**Figure 5:**
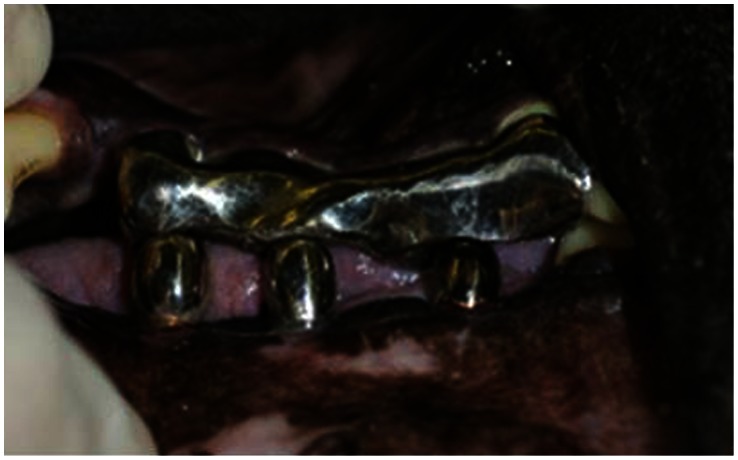
Measurement of vertical marginal bone loss around implant (MBL). **(**1) IR: Implant length of Radiograph (perpendicular distance from the implant shoulder to the most apical aspect of the implant.) and (2) MBLR: Radiographically evaluated MBL. MBLRm is the average perpendicular distance from implant shoulder to first visible apical bone-to-implant contact at the mesial side. MBLRd is the same at the distal side. MBLR is calculated by the average value of MBLRm and MBLRd.

Radiological measurements were performed using the following landmarks [13] : (1) IR, Implant length of radiograph (perpendicular distance from implant shoulder to the most apical aspect) and (2) MBLR, vertical marginal bone loss around the implant of radiograph (average perpendicular distance from the implant shoulder to the first visible apical bone-to-implant contact in the mesial (MBLRm) and distal (MBLRd) aspects of the implant) (Fig. 3). The measurement values of vertical marginal bone loss around the implant (MBL) were calculated using the actual implant length (I) and the following ratio: I/MBL = IR/MBLR. Changes of MBL at 12 and 24 weeks after implant placement were measured (Fig. 2). Ten weeks after implant placement (10 Weeks), a second operation was performed to attach healing abutments to the implants. After further 2 weeks (12 Weeks), superstructures composed of a gold–silver–palladium alloy (8 mm of height; Castwel®M.C., GC, Japan) were constructed using an articulator. These were attached to the abutments but not interconnected (Fig. 4). The occlusal plates were attached to the maxillary molars to maintain occlusal contact with the superstructures. Occlusal adjustment was done using physiological forces and evaluated by checking the position of the remaining teeth. (Fig. 5). After superstructure attachment (At 12 Weeks), immediately 25 mg/kg of fluorescent dye (calcein green; Sigma Chemical Co., St. Louis, MI, USA) [14] was intravenously injected to evaluate bone remodeling (Fig. 2). Following superstructure attachment, the animals were fed a hard pellet diet, and oral hygiene procedures were performed 5 times per week with 100 ml of 0.05% chlorhexidine gel [15] (Concool Gel®; WellTech Co., Japan) to clean peri-implant mucosa and gingiva of residual teeth.

**Figure 6:**
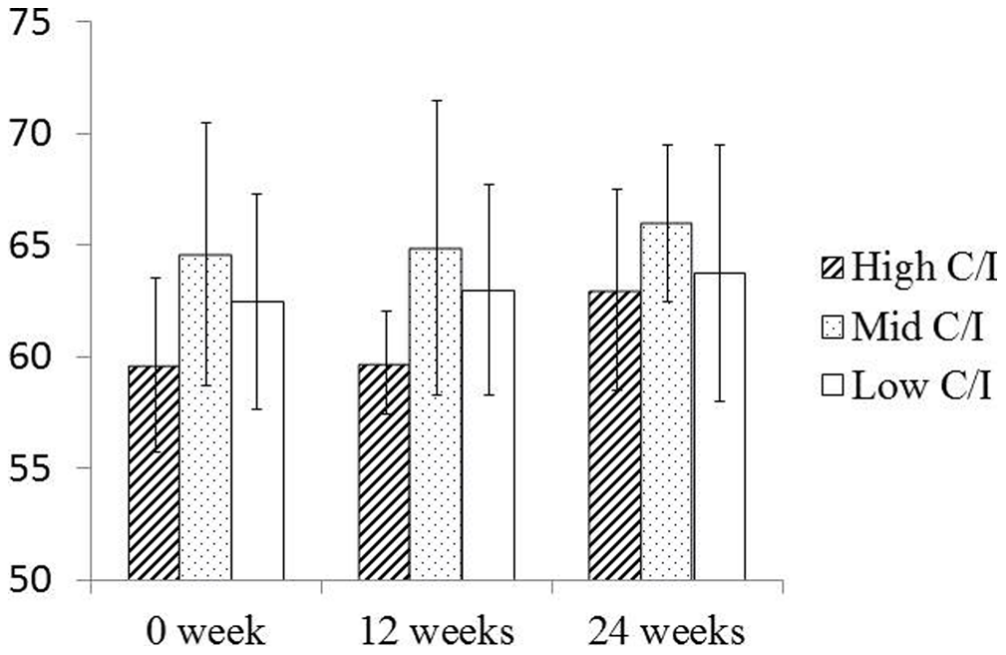
Region under observation. The region under observation extends from buttom of the threads of the implant to1 mm distance at both mesial and distal sides.

Twenty-four weeks after implant placement (At 24 Weeks), ISQ measurement was done and radiographic pictures were taken. Then the animals were sacrificed and bone blocks containing the implants were resected. The blocks were then immersed into 10% neutral formalin for 48 h, dehydrated several times with alcohol, and embedded in photopolymerized methacrylate resin (Technovit® 7200VLC; Exakt Apparatebau, Kulzer, Hamburg, Germany) under reduced pressure. Non-decalcified resin sections were obtained using Hard Tissue Cutting Machine BS-5000 (Exakt Apparatebau, Kulzer, Hamburg, Germany) and an ultra-precision hard tissue grinder (Microgrinding machine MG-4000, Exakt Apparatebau, Kulzer, Hamburg, Germany). Non-decalcified ground mesio-distal cross-sections of approximately 70 µm thickness at the center of the implant were then prepared. Remodeling activity was observed using a fluorescent microscope (AX-70-Macro; Olympus Co., Tokyo, Japan) for a distance of 1 mm on both mesial and distal aspects (Fig. 6). The sections were then stained with toluidine blue and examined by light microscopy (AX70-Macro). Microscope images taken using a digital camera (DP71, Olympus Co.) were uploaded to a personal computer (Dimension 5150C; Dell Inc., TX, USA), and histomorphometry was performed using imaging analysis software (Image J; National Institutes of Health, Bethesda, MD, USA). All values were statistically analyzed by one-way layout analysis of variance and multiple comparison, with the *p* level set at 5%.

## Results

All implants remained immobile during the experiment and no inflammatory reaction was detected around the peri-implant mucosa in all 3 groups throughout the observation period. No mobility or displacement of any implant was noted and no mechanical complications occurred.

### ISQ Values

**Figure 7:**
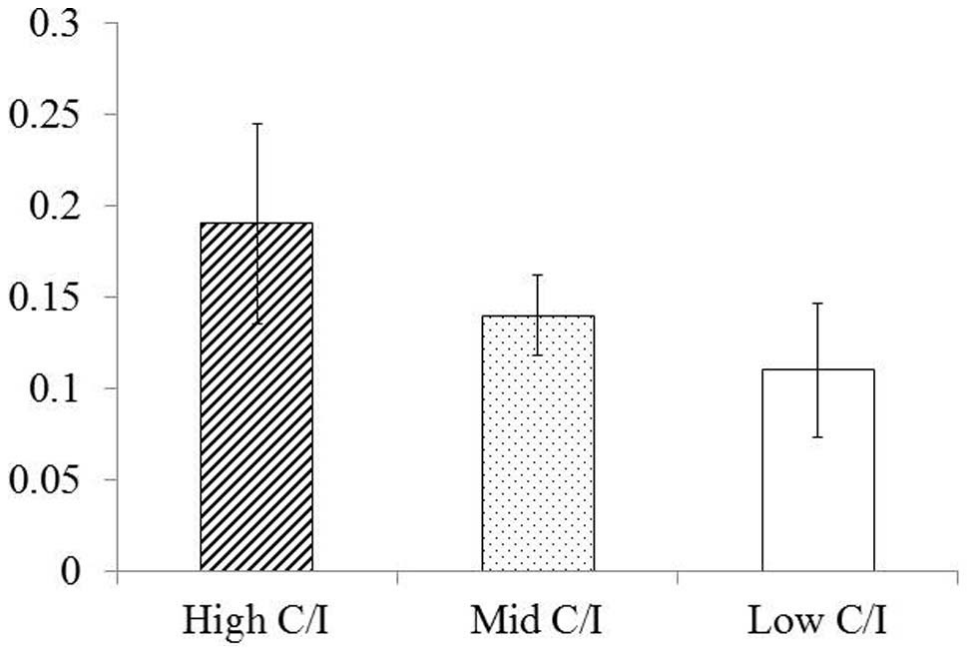
ISQ values at 0, 12, and 24 weeks. Lowest ISQ values are shown in High C/I group. Values are increased with time in all 3 groups, however there is no significant difference among 3 groups at each week (*p*>0.05).

The mean ISQ values of all 3 groups at 0, 12, and 24 weeks are shown in Fig. 7. Values in the High C/I group were 59.63±5.88, 59.75±6.60, and 63±3.49 respectively. Values in the Mid C/I group were 64.63±4.82, 64.88±4.73, and 66±5.71. Values in the Low C/I group were 63.13±3.92, 62.5±2.27, and 63.75±4.5. Values increased with time in all 3 group, without any significant correlation with changing C/I values (*p>*0. 05).

### Radiological Evaluation

**Figure 8:**
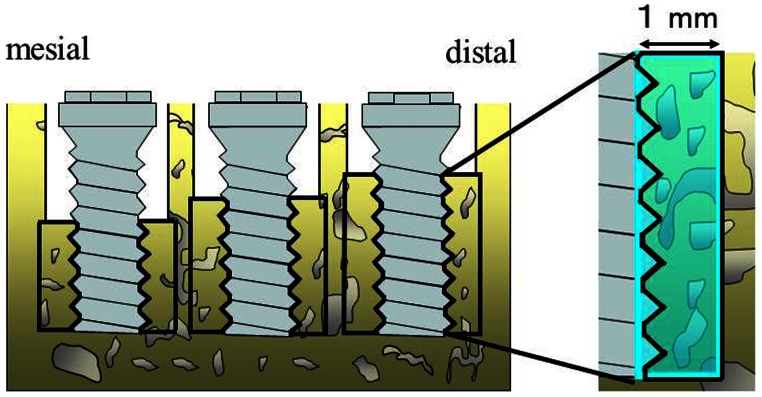
Changes of MBL between 12 and 24 weeks. Highest change is shown in High C/I group, however MBLs in different C/I groups do not show any significant difference (*p*>0.05).

Mean MBL in the High, Mid, and Low C/I groups was 0.19±0.99, 0.15±0.33, and 0.11±0.53 mm, respectively, between weeks 12 and 24.Changes of MBL in all 3 groups ranged between 0.11 and 0.19 mm, showing no significant difference (Fig. 8) (*p>*0.05).

### Histological Changes

**Figure 9:**
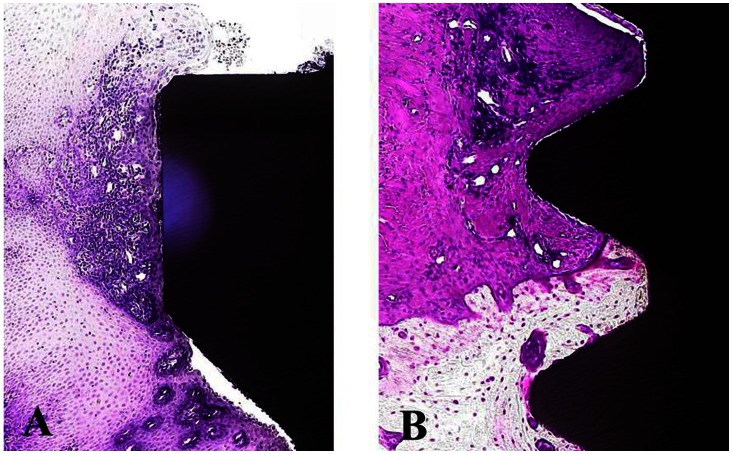
(A) Inflammatory reaction was observed only in the crevicular epithelium. (B) No bone destruction was caused by inflammatory changes.

**Figure 10:**
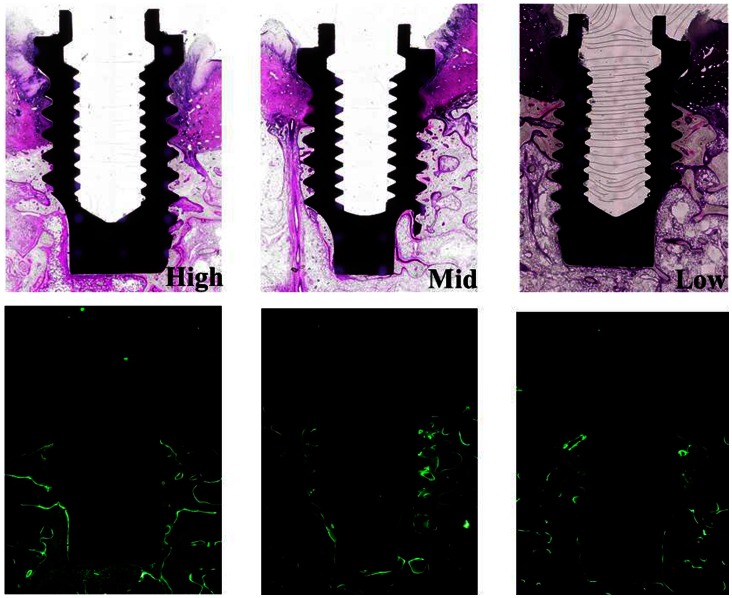
Histological specimen of High, Mid and Low C/I at 24 weeks. All implants in 3 groups are well osseointegrated (Upper side). Broad active remodeling is detected in High C/I group with more fluorescence-labeled bone than in Mid C/I and Low C/I groups (Lower side).

Direct bone contact was detected in all implants in the High, Mid and Low groups, and no soft tissue intervention was seen between bone and implant. Inflammatory reaction was observed only in the crevicular epithelium. However, no bone destruction was caused by inflammatory changes, and no difference in the degree of inflammatory reaction was noted among groups (Fig. 9A, B). Fluorescence-labeled bone was widely detected in all 3 groups, indicating active remodeling, though this was seen more in the High than in the Low C/I group (Fig. 10).

## Discussion

The findings from this study demonstrate that an excessive increase in C/I ratio under appropriate plaque control induced active bone remodeling while exerting no effect on marginal bone loss. Therefore, our hypothesis that excessively increased C/I under appropriate plaque control maintains favorable implant stability has been demonstrated by the fact that mean ISQ values under High C/I yielded a positive outcome, and these findings are supported by histological evidence that High C/I increasing stress at peri-implant bone induced high remodeling activity. Therefore, it may be considered that high remodeling activity prevent MBL and provide favorable implant stability under proper plaque control.

It has become common in recent years to evaluate implant stability by measuring ISQ values clinically [9]–[11]. To examine the relationship between implant stability and ISQ values, ISQ values are measured continuously. In this study, no significant difference in bone resorption was observed between High and Low C/I groups. Direct contact with bone was maintained around all implants. These results are in agreement with those from a study by Tawil *et al*. [8] showing no correlation between C/I ratio and marginal bone loss. This finding indicates that ISQ values might not be an indicator of marginal bone loss. ISQ values in our study ranged between 60 and 68, which concurs with a report on 16 human implants by Degidi *et al.* [16] who found a statistically insignificant correlation between ISQ values and mineralized bone–implant contact percentage. In the present study, changes in MBL in all 3 groups ranged between 0.11 and 0.19 mm, showing no significant difference. The mean ISQ value for all 3 groups at 0, 12, and 24 weeks exceeded 47. Since Nedir *et al*. [12] reported that implant stability can be reliably confirmed for implants displaying an ISQ of >47, we concluded that all implants in this study were stable.

Despite implant treatment having demonstrated a high success rate, implants are still susceptible to peri-implant infections, i.e., mucositis and peri-implantitis [17]. Peri-implant mucositis is an inflammatory lesion characterized by redness and swelling of the soft mucosal tissue, whereas peri-implantitis is often associated with suppuration and deepened pockets, and is always accompanied by peri-implant bone resorption [18], [19]. Marginal bone loss increases the C/I ratio and is considered one of the geometric load factors that may increase the risk of mechanical complications [20]. Osseointegration is defined as “the formation of a direct interface between an implant and a bone with normal remodeling,” [21], and therefore, C/I ratio is considered a major complicating factor in peri-implantitis. Treatment for peri-implantitis as described by Mombelli *et al.* [22] involves attempting to stop progression as early as possible by removal of bacterial deposits. In that study, in order to maintain healthy peri-implant mucosa, and gingiva of residual teeth brushing with chlorhexidine was performed 5 times per week. This prevented the establishment of peri-implantitis, and histologically, there was no evidence of inflammatory cells in the peri-implant soft tissue or peri-implant radiolucency. Our animal model was therefore designed to permit removal of bacterial deposits in order to prevent the development of peri-implantitis.

In this study, we created 3 types of vertical bone loss to simulate C/I situations (High C/I, 4 mm; Mid C/I, 3.25 mm; Low C/I, 2 mm). Several studies [15], [23] have shown no detrimental effects with C/I ratios of 1∶1.75 and 1∶2; therefore, vertical bone loss of 2 mm was defined as Low C/I for a C/I ratio of 1∶2. Schulte *et al.* [24] reported the interesting finding of a high survival rate (98.2%) seen after a mean (SD) follow-up period of 2.3 (1.7) years. This high survival rate also suggests that the C/I ratio is dissimilar to the crown-to-root ratio of natural teeth when determining prognosis. Gentile *et al.* [25] estimated the survival rate of short Bicon dental implants (5.7 mm in length) and compared this with implants of greater length (≥8 mm), finding no difference in survival rates. An assumption could be made that shorter implants have a larger C/I ratio than longer implants, yet no difference in survival rate was noted. Rokni *et al.* [23] reported that values for C/I ratios relate to the degree of marginal bone loss. That study included 198 implants in which the average (SD) C/I ratio was 1.5 (0.4), with a range of 0.8∶1 to 3∶1. Calculation of the C/I ratio was based on the measurement of articulated diagnostic casts. The authors reported no association between C/I ratio and degree of marginal bone loss. These C/I ratios are similar to those from the present study, though different methods were used to calculate them.

No mechanical complications occurred with any implant, and no loosening of hexagonal screws was noted during the observation period. Nissan et al. [26] reported that a related parameter is crown height space (CHS), defined as the distance from the crest of the alveolar bone to the plane of occlusion. The biomechanics of CHS are related to lever arm mechanics. These workers concluded that prosthetic failure occurred at a C/I ratio of 1∶1.75 or greater and a CHS of 15 mm or greater. We used superstructures of 8 mm height in the present study, and our results support those described above.

In the present study, more fluorescence-labeled bone areas were detected in the High C/I group than in the Low C/I group. It has been reported that a force greater than normal loading but within the biologically acceptable range promotes bone formation and stabilization at a high level of bone mass [27], [28]. Gomez-Polo et al. [29] reported that an increased C/I ratio increased the mechanical overload on bone and caused cellular alterations; this has been referred to as remodeling. It is thought that excessive remodeling activity prevents MBL and provides favorable implant stability under appropriate plaque control. Our findings suggest implant stability can be maintained favorably despite High C/I under appropriate plaque control. As a result, excessive increased C/I is in function in observation period. However, in this study, healthy peri-implant mucosa was maintained throughout the experiment, and a generalized limitation of the canine model is that no lateral mandibular movement occurs. It is important to not only control inflammation but also regulate lateral loading with excessively increased C/I.

### Conclusion

These findings suggest that increased C/I may not be a risk factor for implant failure if the peri-implant mucosa is kept healthy, as observed in the animal model in this study.
